# The Roles of Skin Fibroblasts at Local Acupoints in Chrono-Acupuncture

**DOI:** 10.1155/2020/3731510

**Published:** 2020-03-25

**Authors:** Nannan Liu, Zhengyu Zhao, Qizhi Zhou, Xiaohong Zhang, Yu Wang, Shiqi Huang, Dingjun Cai

**Affiliations:** ^1^School of Acupuncture and Tuina, Chengdu University of Traditional Chinese Medicine, Chengdu, Sichuan, China; ^2^Xinjin Hospital of Traditional Chinese Medicine, Chengdu, Sichuan, China; ^3^Xiangyang Hospital of Traditional Chinese Medicine, Xiangyang, Hubei, China; ^4^Zhizhentang Traditional Chinese Medicine Clinic, Chengdu, Sichuan, China

## Abstract

**Objective:**

The aim of this study was to demonstrate the peripheral mechanisms of chrono-acupuncture by observing acupuncture at different time points affecting relative proteins to regulate the cytoskeleton of fibroblasts differently.

**Methods:**

A total of 108 male SD rats (180–220 g) that have basic pain threshold within 3–10 s were selected and randomly divided into group A (*n* = 72) and control group (*n* = 36). After the succession of modeling with CFA injection, the rats in group A were randomly divided into model group and acupuncture group, each group containing 36 rats. Then according to the different treatment time, each group was randomly classified into 6 subgroups (ZT0, ZT4, ZT8, ZT12, ZT16, and ZT20), each subgroup containing 6 rats (*n* = 6). On the second day of successful modeling, the rats in the acupuncture group received acupuncture treatment at the corresponding time point, while the control group and the model group were only tied up at the corresponding time point without any treatments. Methods of operation: use 0.5-inch needles, puncture the rats' “Zusanli” on the affected limb, with Twirling manipulation for a minute after every five minutes; the treatment lasts thirty minutes in total. After 7 days of treatments, the skin and subcutaneous tissue of rats' acupoint area of “Zusanli” on the affected limb were taken and then stained by immunofluorescence double staining method to observe the expression of the fibroblast cytoskeleton F-actin and *β*-tubulin under the LSCM while using western blot to observe the expression of P38MAPK/P-P38MAPK.

**Results:**

The expression of the cytoskeleton F-actin and *β*-tubulin at acupoint area in the acupuncture group was significantly higher than that in the control and model group. The effect of acupuncture on the restructure of the fibroblast cytoskeleton is different at different time points, the most effective time point was at ZT12 while the least at ZT16. Acupuncture can decrease the high expression of P-P38MAPK/P38MAPK in the model group, and the effect has time differences. The expression of P-P38MAPK/P38MAPK increased more significantly at ZT16 than ZT12.

**Conclusion:**

The remodeling difference of fibroblast cytoskeleton after receiving acupuncture treatment could be one of the peripheral bases of the chrono-acupuncture.

## 1. Introduction

Circadian rhythms are daily oscillations in behavior and physiology that prepare organisms to better react to and anticipate changes in the environment. These cellular clocks are conserved from cyanobacteria to mammals and are accordingly found in almost every cell in multicellular organisms that evolved from unicellular progenitors [1]. A zeitgeber is an external cue that entrains or synchronizes the biological rhythms to the earth's cycle [2]. The circadian clock can be entrained by the external environment. To maintain clock-environment synchrony, zeitgebers induce changes in the concentrations of the molecular components of the clock to levels consistent with the appropriate stage in the 24-hour cycle, a process termed “entrainment” [3]. Many different diseases have shown distinct circadian patterns, either in the symptoms or in the clinical data. Various pain syndromes not only show circadian rhythm but also affect the circadian rhythm of the body. Many patients with rheumatoid arthritis (RA) present a circadian rhythm in the severity of symptoms, with a significant worsening in the morning. Previous studies have shown that the change of pain threshold has an obvious circadian rhythm [4, 5].

**Figure 1:**
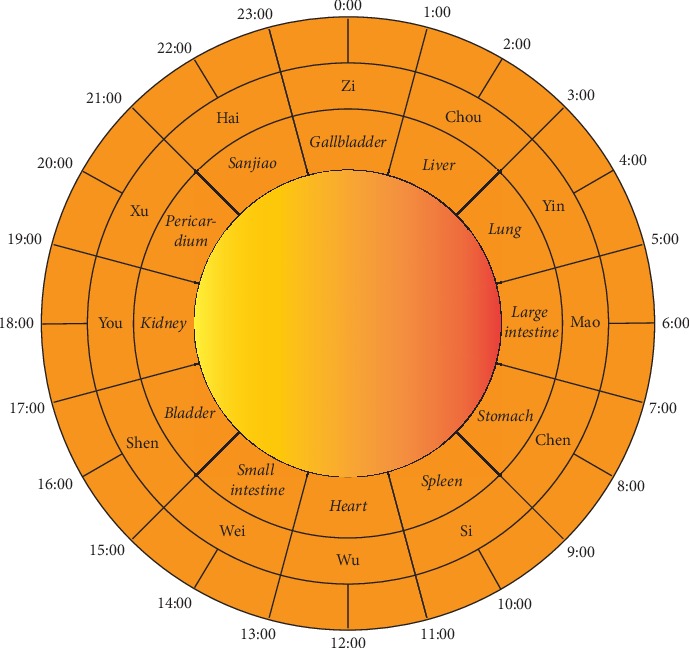
Diagram of organs related to time in TCM.

Chrono-acupuncture, which has been used for centuries, is thought to be potentially helpful for many diseases. The Traditional Chinese Medicine considers each of the twelve regular meridians has a special 2-hour slot in the 24-hour clock, as well as the corresponding organs (Figure 1). Chrono-acupuncture carries out the treatment based on the rhythm of qi and blood flow in the meridians and organs. A study to verify the efficacy of selective-time-acupuncture (9:00–11:00 am) on chronic fatigue syndrome has shown that the improvements in the scores of fatigues (both physical and mental) of the selective-time-acupuncture group were superior to the acupuncture group [6]. Acupuncture significantly increased nocturnal diastolic blood pressure (BP) dipping and decreased nighttime diastolic BP [7]. Electroacupuncture has regulation effects on the circadian rhythm of temperature and melatonin in depression rat model [8]. Our previous study has demonstrated that puncturing “Zusanli” acupoint has significant effect on relieving pain, and this effect has time difference; acupuncture at “Zusanli” acupoint can most increase rats' pain threshold at ZT12 [9]. Thus, the curative effect of acupuncture at different time points was different.

There have been studies showing through fMRI that reactions in the brain are different between puncturing acupoint and nonacupoint, acupuncture and sham acupuncture. Thus, acupoints have got specificity [10]. Through the measurement of the local volt-ampere characteristics of acupoints, it was found that, under physiological conditions, the “Yuan” acupoints of the human body have a circadian rhythm, which changes in the inertial area of the acupoints [11]. Acupuncture at “Zusanli” acupoint can promote the increase of the number of local mast cells in the acupoints and increase the degranulation of the cells. The histamine and substance P released by mast cells can promote the enhancement of local vascular permeability and promote the formation of tissue fluid and corresponding increased lymphatic reflux [12].

A fibroblast is a type of cell that synthesizes the extracellular matrix and collagen [13]. Fibroblasts are the most common cells of connective tissue in animals. Fibroblasts have a branched cytoplasm surrounding an elliptical, speckled nucleus having two or more nucleoli. They are not restricted by a polarizing attachment to a basal lamina on one side. Fibroblasts make collagens, glycosaminoglycans, reticular and elastic fibers, glycoproteins, and cytokine TSLP [14]. Tissue damage stimulates fibrocytes and induces the mitosis of fibroblasts. The twisting method of acupuncture can cause contraction of the fibroblasts which can further pull the collagen and cause the deformation of the matrix, finally transmitted to the entire connective tissue by the mechanical force signal generated. Therefore, fibroblasts are the main response cells to the mechanical force of acupuncture. Like neurons in the suprachiasmatic nucleus (SCN), the master circadian pacemaker in the brain, single fibroblast can function as independent oscillators [15]. The purpose of this study is to demonstrate the peripheral mechanisms of chrono-acupuncture by observing acupuncture at different time points affecting relative proteins to regulate the cytoskeleton of fibroblasts differently.

## 2. Methods

### 2.1. Animals

A total of 108 clean graded male SD rats (provided by the animal center of Sichuan provincial hospital, permit number: SCXK 2013-15) that weighed 180–220 g were raised in isolated units under 12 h : 12 h light/dark cycles (lights on at 7:00, ZT0 and off at 19:00, ZT12) for rhythm domestication for 7 days before the experiments. All rats had free access to water and food during whole experiment. Animal care was carried out in accordance with the Instruction for Ethical Treatment of Animals issued by the Ministry of Science and Technology, China, 2006. We tried to minimize the number and suffering of the laboratory animals. All procedures and animal experiments were approved by the Animal Care and Use Committee of Chengdu University of Chinese Medicine (China).

### 2.2. Assessment of Pain Threshold

The pain threshold of rats was tested by the tail-flick method before and after treatment. Standard rats which flicked between 3 and 10 seconds on the test were involved in the experiment. The pain threshold was finished within 1 hour under the same conditions. In addition, in order to avoid scalding the rats and calm them down, each one was measured at intervals of 5 minutes or more.

### 2.3. Grouping and Modeling

36 rats were randomly selected from the 108 rats as control group and were subcutaneously injected with 0.1 ml 0.9% saline into the right hind paw 1 day before the experiment, while the remaining 72 rats were subcutaneously injected 0.1 ml complete Freund adjuvant (CFA) into the right hind paw 1 day before the experiment under the same conditions as group A. The rats established by CFA should be in acute pain stage after 24 hours of injection and the syndromes showed that the time of tail-flick shortened accompanied with red-swollen paw. After successful replication of the model, they were randomly divided into the model group (*n* = 36) and the acupuncture group (*n* = 36) based on the pain threshold. Each group was then randomly divided into 6 subgroups (ZT0, ZT4, ZT8, ZT12, ZT16, and ZT20, *n* = 6), respectively.

### 2.4. Treatment

On the second day of successful modeling, rats in the acupuncture group received acupuncture treatment at the corresponding time points. The “Zusanli (ST36)” acupoint on the affected limb was punctured with a minute of Twirling manipulation after every 5 minutes for 30 minutes each day and for 7 days in total, while the control group and model group were tied up exactly the same way as the acupuncture group without any treatment at the corresponding time point.

### 2.5. Immunofluorescent

At the end of all treatment, the skin and subcutaneous tissue of “Zusanli (ST36)” in the affected side (size: 5 mm ∗ 5 mm, depth:2-3 mm) were removed, wrapped in 5 cm ∗ 5 cm foil paper and put in liquid nitrogen immediately. The frozen tissue was then removed and sectioned using a semiautomatic cryosection machine (with a slice thickness of 6–10 *μ*m) and allowed to air-dry and then immunofluorescence double-labeled. Confocal laser microscopy was used to collect images for each slice. Image-Pro Plus 6.0 image analysis system was used to determine the integrated optical density (IOD) and area (Area) of all images, and the average optical density (mean density, MD) was calculated.

### 2.6. Western Blot

Based on the former experimental results, the further experiment was conducted at the peak phase (ZT12) and the valley phase (ZT16). 36 SD male rats were needed in this part of the experiment. The methods of assessment of pain threshold, treatment, modeling, and grouping were the same as the former. At the end of all treatment, the skin and subcutaneous tissue of “Zusanli (ST36)” in the affected side (size: 5 mm ∗ 5 mm, depth: 2-3 mm) were removed, wrapped in 5 cm ∗ 5 cm foil paper, and put in −80°C liquid nitrogen immediately. Western blot was used to determine the expression of P38MAPK/P-P38MAPK. Scanning analysis was performed using a gel image analysis imaging system and the results were expressed as relative expression levels of the target protein. Relative expression level of target protein = integral optical density (IOD) of target protein/integral optical density (IOD).

### 2.7. Statistical Analysis

All data were analyzed using SPSS 23.0 for one-way ANOVA, and results were presented as mean ± standard deviation (SD). RMANOVA and LSD test were used to compare between the groups; significance was determined at *P* < 0.05. Linear regression was used to analyze inter-indication correlation; *P* < 0.05 was considered statistically significant.

## 3. Results

### 3.1. Time Has a Significant Influence on the Pain Threshold as well as the Therapeutic Effect of Acupuncture

**Figure 2:**
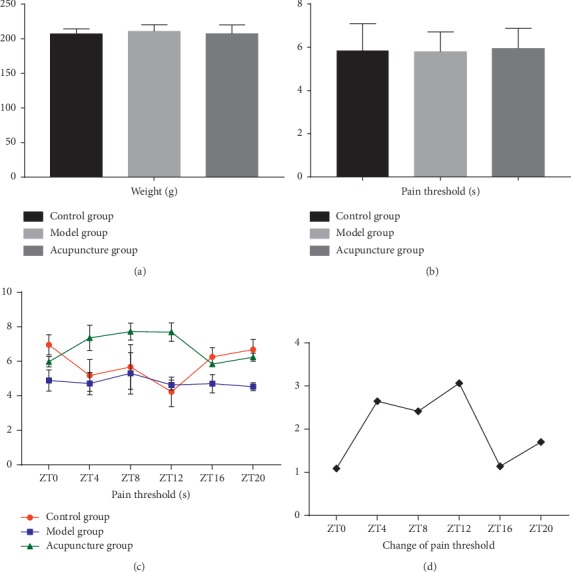
(a) There is no significant difference among the baseline weight of all three groups (*n* = 36) (*P* > 0.05). (b) No significant difference of the baseline pain threshold among all three groups (*n* = 36) (*P* > 0.05). (c) Pain threshold at different time points of different groups (*n* = 6); there is a rhythm pattern and CFA injection and acupuncture can affect the rhythm pattern. (d) Change of pain threshold after acupuncture compared with the model group was calculated at different time points; acupuncture is most effective at ZT12, while least effective at ZT0 and ZT16 (*n* = 6).

The baseline weight and pain threshold had no significant difference between all three groups (Figures 2(a) and 2(b)). Time factor had significant influence on the pain threshold in the control group (*P* < 0.05), with peak value at ZT0, and valley value at ZT12. This rhythmic pattern of pain threshold changed significantly after modulization; time had no significant effect on the pain threshold of the model group (Figure 2(c)). Acupuncture at “Zusanli” acupoint can increase the pain threshold of the model group significantly, and time is an influential factor, most effective at ZT12 and least effective at ZT0 and ZT16 (Figure 2(d)). As a result, the acupoint reaction has a temporal dynamic change, and the strength of this change is affected by time factors.

### 3.2. The Effect of Chrono-Acupuncture on the Fibroblast Skeleton

**Figure 3:**
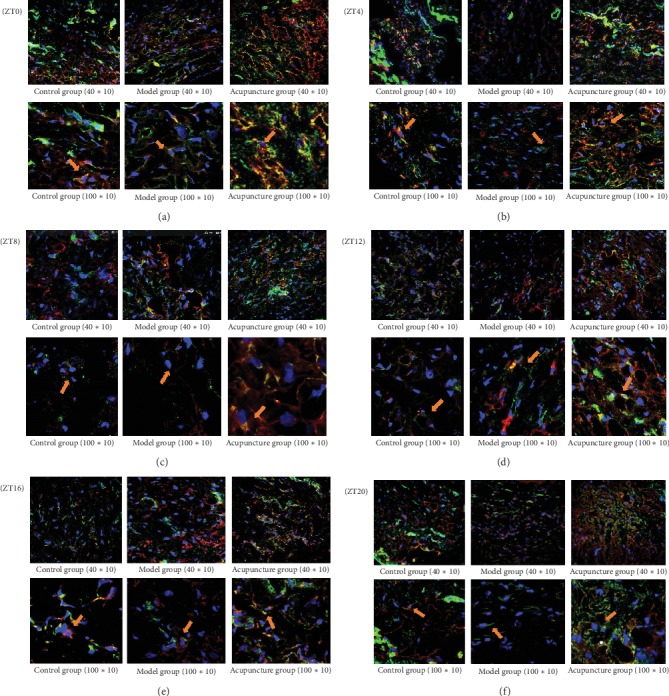
The immunofluorescent stain of fibroblasts at the skin of acupoint area, red light (vimentin), green light (F-actin), and blue light (DAPI). Acupuncture can change the shape and arrangements of fibroblasts, and time has an influence on this change. (a–f) Pictures of immunofluorescent stain of fibroblasts at different time points (*n* = 6).

The mechanical force generated by acupuncture can significantly change the shape and arrangement of fibroblasts at acupoint area. The fibroblasts were arranged more closely with fusiform cytoskeleton and increased cross-sectional area. The fibroblasts were connected with the skeleton of adjacent cells and arranged in a filamentous or net shape. The stain was also more intense, with more overlapping of red light (vimentin) and green light (F-actin) which appeared orange-red (Figure 3). Time is also an influential factor in these changes. The change was most obvious and significant at ZT12 (Figure 3(d)) with round-shaped fibroblasts, increased cross-sectional area, and more intense staining (orange-green color), the cytoskeleton is evenly distributed around the nucleus, and the intercellular skeleton was interwoven into a network. The effect of acupuncture on the skeleton of fibroblasts was least significant at ZT16 (Figure 3(e)), with not very closely related cytoskeleton and slightly increased cross-sectional area.

### 3.3. The Effect of Chrono-Acupuncture on the Expression of F-Actin and *β*-Tubulin in Rats' Skin of “Zusanli (ST36)” Acupoint Area

**Figure 4:**
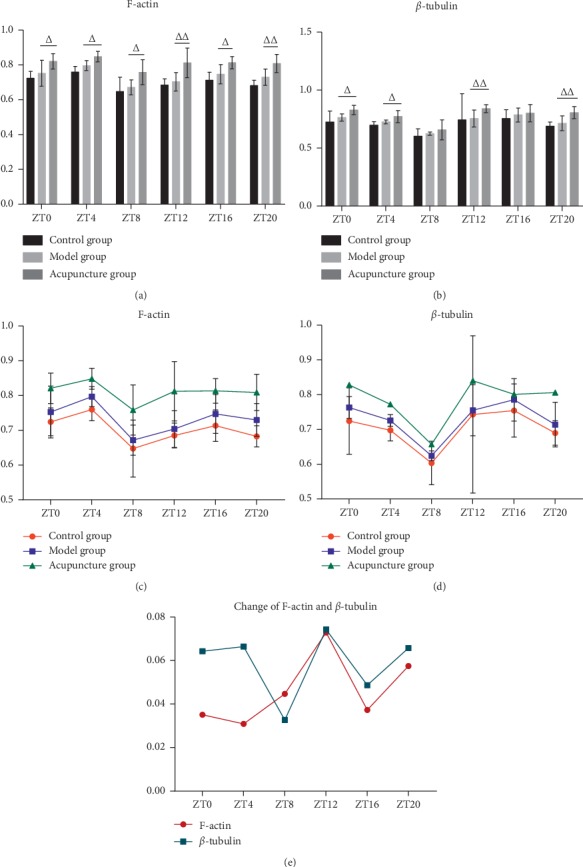
Acupuncture can significantly increase the expression of both F-actin and *ß*-tubulin with time differences. ^△^(*P* < 0.05). ^△△^(*P* < 0.01). (a) Difference of average F-actin optical density between control group, model group, and acupuncture group (*n* = 6). (b) Time pattern of average optical density of F-actin (*n* = 6). (c) Difference of average *β*-tubulin optical density between control group, model group, and acupuncture group (*n* = 6). (d) Time pattern of average optical density of *β*-tubulin (*n* = 6). (e) The change of expression of F-actin and *β*-tubulin between acupuncture group and model group; acupuncture was most effective at ZT12 on the expression of F-actin and *β*-tubulin (*n* = 6).

For all time points, compared with the control group, the expression of F-actin in model group has no significant changes (*P* > 0.05), while compared to the model group, the expression of F-actin in the skin increased significantly (*P* < 0.05) (Figure 4(a)). In control group, the expression of F-actin peaked at ZT12 and reached bottom at ZT8, which indicates that the time factor can affect the expression of F-actin. The expression of F-actin in the model group and acupuncture group also peaked at ZT12 and reached the bottom at ZT8, which indicates that there is no significant impact of modeling or acupuncture on the rhythm pattern of the expression of F-actin in the acupoint area (Figure 4(b)). However, compared with model group, the expression of F-actin in the acupoint area in the acupuncture group has significantly increased, the effect of acupuncture peak at ZT8 and ZT20 (Figure 4(e)).

Compared with the control group, the expression of *β*-tubulin in the model group has no significant changes (*P* > 0.05); compared with the model group, the expression of *β*-tubulin in the acupuncture group increased at all time points (Figure 4(c)). Time is an influential factor for the expression of *β*-tubulin, and the pattern appeared bimodal, which peaked at ZT0 and ZT16 for both the control group and model group; the valley value appeared at ZT8. However, the acupuncture group showed a trimodal distribution with peak values appearing at ZT0, ZT12, and ZT20, and the valley value appearing at ZT8 (Figure 4(d)). Acupuncture at different time points has different effects on the expression of *β*-tubulin with the most significant influence at ZT12 and the least at ZT8 (Figure 4(e)).

The linear regression analysis was used to analyze whether the expression of F-actin in fibroblasts was affected by the expression of *β*-tubulin. The results showed that the expression of *β*-tubulin had a significant effect on the expression of F-actin and was positively correlated.

### 3.4. The Effect of Acupuncture at Different Time Points on the Expression of P-P38MAPK/P38MAPK

**Figure 5:**
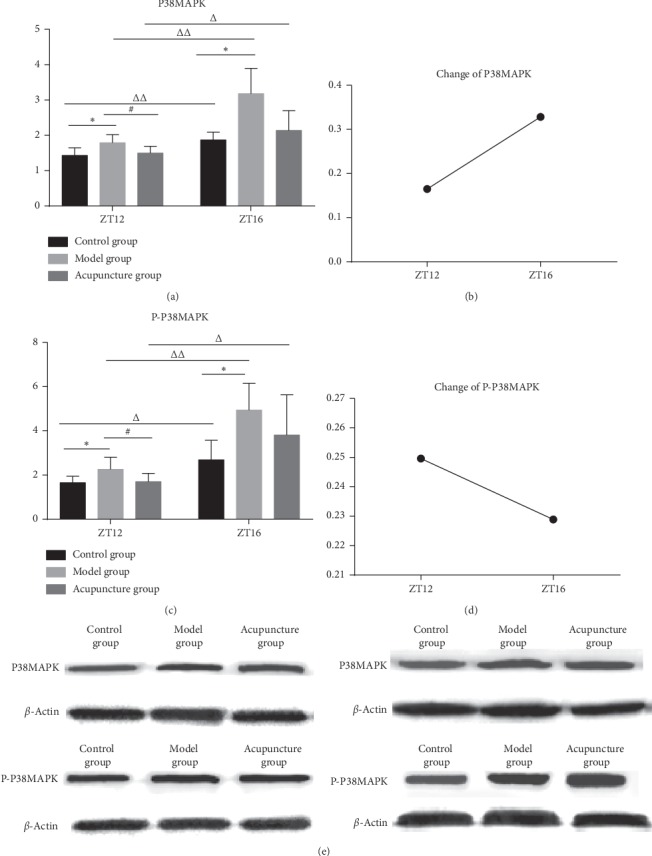
The expression of P38MAPK and P-P38MAPK at skin of acupoint area. There is statistical difference between control group and model group (^*∗*^*P* < 0.05). There is statistical difference between acupuncture group and model group (#*P* < 0.05). There is statistical difference between ZT12 and ZT16 (^△^*P* < 0.05, ^△△^*P* < 0.01). (a) The average optical density of P38MAPK at ZT12 and ZT16 in all three groups (*n* = 6). (b) The change of expression of P38MAPK after acupuncture is more significant at ZT16 (*n* = 6). (c) The average optical density of P-P38MAPK at ZT12 and ZT16 in all three groups (*n* = 6). (d) The change of expression of P-P38MAPK after acupuncture is more significant at ZT12 (*n* = 6). Expression of P38MAPK at ZT12. Expression of P38MAPK at ZT16. Expression of P-P38MAPK at ZT12. Expression of P‐P38MAPK at ZT16. (e) Expression of P38MAPK and P‐P38MAPK at ZT12 (left); expression of P38MAPK and P‐P38MAPK at ZT16 (right).

We select the time points at which the analgesic effect of acupuncture is the most (ZT12) and least (ZT16) significant. Western Blot was used to determine the expression of P38MAPK and P-P38MAPK in the skin of acupoint area (Figure 5(e)). Acupuncture can decrease the high expression of P-P38MAPK/P38MAPK in the model group, and the effect has time differences. At ZT12, the expression of P-P38MAPK/P38MAPK is significantly increased in the model group compared with the control group, and the expression of P-P38MAPK/P38MAPK is significantly decreased in the acupuncture group compared to the model group. At ZT16, compared with the control group, the expression of P-P38MAPK/P38MAPK is significantly increased in the model group. In all three groups, the expression of P-P38MAPK/P38MAPK increased more significantly at ZT16 than ZT12 (Figures 5(a) and 5(c)). Acupuncture can affect the expression of P38MAPK more significantly at ZT16 and P-P38MAPK at ZT12 (Figures 5(b) and 5(d)).

## 4. Discussion

Cells sense biochemical, electrical, and mechanical cues in their environment that affect their differentiation and function. Mechanical signals, unlike biochemical and electrical signals, can propagate with the diffusion of proteins or ions. Forces are transmitted through mechanically stiff structures, for example, through cytoskeletal elements such as microtubules or filamentous actin. Mechanical forces are inherent in the cellular environment; force is a signal that cells must take advantage of to maintain homeostasis and carry out their functions [16]. The sensory device in the acupoint area can transform different stimuli into transducer potentials or directly generate afferent nerve impulses; after acupuncture, acupoints form extremely complex biological information flows, such as nerve signals, body fluids, and energy [17]. The mechanical force generated by acupuncture stimulates the acupoint area and responds mainly through the cytoskeleton. Fibroblasts, as a large number of cells in the subcutaneous loose connective tissue, are very sensitive to changes in their local tension. The stretching of the subcutaneous tissue can cause a transient increase in local tension and activate fibroblasts to reconstitute their cytoskeleton [18]. “Zusanli” acupoint is the He-sea point of the Stomach Meridian of Foot-Yangming. The fascia tissue cells in “Zusanli” acupoint and its adjacent area are mostly fibroblasts. The mechanical stimulation on local area significantly accelerated the synthesis and release of PGE_2_ and IL-6 [19]. Fibroblasts at acupoint area can directly receive mechanical stimulation, and then the mechanical signals were transformed as biological signals.

Time appears as a factor that affects the cytoskeletal reconstruction, with the most significant change at ZT12 after acupuncture and least significant change at ZT16. This indicates that the same amount of force at different time points can have different effects, which is also obviously shown from the change in pain thresholds. Time factor had a significant effect on the basic pain threshold of the rats and showed rhythmic fluctuations, with a double-valley structure, in which the valley values were at ZT4 and ZT12, and the peak value was at ZT20. The pain threshold of rats changed after modeling with Freund's adjuvant. The highest point appeared at ZT8, and the lowest point was at ZT16. After acupuncture treatment, the pain threshold of rats was higher than that of the model group, with the most significant change at ZT8 and least significant change at ZT16.

The change in the expression of skeleton proteins can affect the reconstruction of the fibroblast skeleton, and therefore the transformation of mechanical force generated by acupuncture was also affected. Actin filaments and microtubules play an important role in cytoskeleton reconstruction. Actin is the material basis of all eukaryotic cell movements. F-actin can be synthesized by the induction of ions such as magnesium, potassium, and sodium ions and adenosine triphosphate [20]. F-actin participates in the movement of cells and cytoplasm, mainly in the form of cell scaffolds to maintain the shape of the cells, as well as the transduction of intracellular signals and the synthesis of various proteins. Changes in the length of F-actin, such as prolongation or shortening, could cause increased or decreased cytoplasmic viscoelasticity. Most of these changes are affected by various types of actin binding proteins [21]. Microtubules are also important in maintaining cell morphology and signal transduction and are mainly composed of several subtypes of tubulin, such as *α*, *β*, *ε*, *γ*, and *δ* as well as many small microtubule-associated proteins (MAPs). Among all subtypes of tubulin, *α*, *β*-tubulin plays an important role in cell migration, differentiation, and other activities, while MAPs are important in the regulation and distribution of microtubules. Both microfilament and microtubules are important parts of the cytoskeletal network system. Changes in the structure of microtubules can affect the activity of F-actin.

In this experiment, fibroblasts were marked by labeling the specific protein vimentin (only fibroblasts express vimentin in the skin) [22]. The morphology of fibroblasts and the changes of F-actin and *β*-tubulin at the acupoint area were observed using confocal laser scanning microscopy. The results showed that the fibroblasts in the acupuncture group were more closely arranged than those in the control and model group, especially at ZT12, and least significantly at ZT16. After acupuncture treatment, the expression levels of F-actin and *β*-tubulin in the skin and subcutaneous tissues at the acupoint area were significantly increased. The expression level of F-actin in the acupuncture group increased most significantly at ZT12 compared with the model group (*P* < 0.01). The expression level of *β*-tubulin was also significantly higher in the acupuncture group than that in the control and model group at ZT12, *P* < 0.01. By collecting the optical density of immunofluorescence of F-actin and *β*-tubulin in fibroblasts, it was found that the expression levels of skeletal proteins of fibroblasts in the control group were different at each time point, suggesting that the expression of fibroblast cytoskeleton proteins was affected by the time factor. The comparison in different groups at the same time point showed that, at ZT8, ZT12, and ZT20, the average optical density of F-actin in the acupuncture group was significantly higher than that in the control and model group (*P* < 0.05); the average optical density of the acupuncture group at other time points was also higher than that of the control and model group, but the difference was not statistically significant (*P* > 0.05). The mean optical density of *β*-tubulin was higher at ZT0, ZT4, ZT12, ZT16, and ZT20 in the acupuncture group than that in the control and model group (*P* < 0.05); the difference was more significant at ZT12 (*P* < 0.01). At ZT8, the average optical density of *β*-tubulin in the acupuncture group was also higher than that in the control and model group, but the difference was not statistically significant (*P* > 0.05). Therefore, the expression of cytoskeletal proteins F-actin and *β*-tubulin at the acupoint area in the acupuncture group was significantly higher than that in the control and model group, especially at ZT12 (*P* < 0.01).

Overall, the efflect of the time factor on the fibroblast F-actin in rat's skin has no statistically significant difference among all three groups, *P*=0.093 > 0.05. However, the time factor has a significant influence on the expression of fibroblast *β*-tubulin in rat's skin (*P*=0.02 < 0.05). By calculating the rate of change of F-actin and *β*-tubulin, we found that acupuncture affects the average optical density of F-actin and *β*-tubulin most significantly at ZT12, and least at ZT4 for F-actin, ZT8 for *β*-tubulin. Therefore, we infer that the effect of acupuncture in fibroblasts is related to F-actin and *β*-tubulin proteins and may involve the regulation of other proteins. At the same time, acupuncture can change the circadian rhythm pattern of fibroblast to a certain extent. Our experiments also showed that the changes in the physiological and pathological states of the skeletal proteins F-actin and *β*-tubulin of rats' skin fibroblasts are not much different, but the production of inflammatory model promotes expression of fibroblasts F-actin and *β*-tubulin to a certain extent. Acupuncture on rat acupoint can increase the expression of the cytoskeletal proteins of fibroblasts F-actin and *β*-tubulin.

Mitogen-activated protein kinases (MAPKs) are a class of serine threonine protein kinases widely distributed in eukaryotic cells. Studies have shown that the MAPK signaling pathway also plays an important role in cytoskeleton regulation. As a classical pathway in the MAPK signaling, P38MAPK kinase induces microtubule multimerization by inhibiting MRP8/MRP14 complex by hyperphosphorylated calcium binding protein MAP14 (S100A9) [23]. P38-MAPK can influence the shape and differentiation of microfilaments by regulating the phosphorylation of keratin closely related to the regulation of fibrillation and the state of keratin cleavage [24]. P38MAPK also regulates cell apoptosis and redifferentiation by affecting the integrity and stability of microfilaments [25, 26]. Following the application of P38/MAPK specific inhibitors to cells, it was found that ADMD significantly inhibited cytoskeletal remodeling, revealing that P38/MAPK plays an important role in the process of cytoskeletal remodeling induced by ADMD. Acupuncture as a mechanical stimulation at “Zusanli” acupoint induces the increase of adenosine in fibroblasts through the activation of the MAPK signaling pathway, ultimately promoting fibroblast proliferation [27].

In this study, we have found that acupuncture treatment can significantly influence the expression of P38MAPK and P-P38MAPK. The inflammatory pain can obviously increase the expression of P38MAPK and its phosphorylation P-P38MAPK in the rats' skin of the acupoint area, and the expression of P38MAPK and P-P38MAPK decreases after acupuncture treatment. The expression of P38MAPK and P-P38MAPK was significantly higher at ZT16 than that at ZT12 in all three groups. Overall, the effect of acupuncture on fibroblast cytoskeleton remodeling is more significant at ZT12 than ZT16, which may be due to the more significant decrease in the expression of P38MAPK at ZT12 after acupuncture treatment, and less significant decrease in the expression of P-P38MAPK at ZT16 after acupuncture treatment.

In conclusion, the effect of acupuncture is inseparable from the changes in the response of the acupoints area. Acupuncture at different time points has different effects on the local fibroblast cytoskeleton remodeling in the acupoints area. This may be due to the difference of the effect on regulating pathways at different time points. It is suggested that the time difference of acupoint reactivity is related to the remodeling of fibroblasts in the acupoints. The reconstruction of fibroblasts after acupuncture may be one of the bases of the acupoint response chronologically.

## Data Availability

The data used to support the findings of this study are available from the corresponding author upon request.

## References

[1] Sarah S. G., Caio T. F., Richard M. S. (2015). Chrono-immunology progress and challenges in understanding links between the circadian and immune systems. *Immunology*.

[2] Grandin L. D., Alloy L. B., Abramson L. Y. (2006). The social zeitgeber theory, circadian rhythms, and mood disorders: review and evaluation. *Clinical Psychology Review*.

[3] Toh K. L. (2008). Basic science review on circadian rhythm biology and circadian sleep disorders. *Annals Academy of Medicine*.

[4] Jankowski K. S. (2013). Morning types are less sensitive to pain than evening types all day long. *European Journal of Pain*.

[5] Costa-Martins J. M., Pereira M., Martins H., Moura-Ramos M., Coelho R., Tavares J. (2014). The influence of women’s attachment style on the chronobiology of labour pain, analgesic consumption and pharmacological effect. *Chronobiology International*.

[6] Ling J. Y., Shen L., Liu Q., Wang L. Y. (2013). Impacts on chronic fatigue syndrome of QI deficiency syndrome and T cell subgroups in patients treated with acupuncture at selective time. *Zhongguo Zhen Jiu*.

[7] Kim H.-M., Cho S.-Y., Park S.-U. (2012). Can acupuncture affect the circadian rhythm of blood pressure? a randomized, double-blind, controlled trial? a randomized, double-blind, controlled trial. *The Journal Of Alternative And Complementary Medicine*.

[8] Yao H. J., Song H. T., Mo Y. P., Zhang T. T., Han X. B., Li Z. G. (2014). Effects of electroacupuncture on circadian rhythm of temperature and melatonin in depression rats model induced by chronic stress. *Zhongguo Zhen Jiu*.

[9] Huang S., Lu X., Shen X. (2019). The roles of extracellular purinergic signaling in local acupoints in chrono-acupuncture analgesia. *Journal of Complement Medicine Alternative Healthcare*.

[10] Campbell A. (2006). Point specificity of acupuncture in the light of recent clinical and imaging studies. *Acupuncture in Medicine*.

[11] Zhou Y., Wang J., Shen X., Jianzi W. (2005). Study on volt-ampere property of meridional points and the prospect of its clinical application. *Shanghai Journal of Acupuncture and Moxibustion*.

[12] Zhang K., Chen B., Zhao X. (2015). Acupoint being amplifier of acupuncture information transmission. *Liaoning Journal of Traditional Chinese Medicine*.

[13] Weissman P., Michaelfry S. (1975). Chick embryo fibroblasts senescence in vitro: pattern of cell division and life span as a function of cell density. *Mechanisms of Ageing and Development*.

[14] Song Y. H., Zhu Y. T., Ding J. (2016). Distribution of fibroblast growth factors and their roles in skin fibroblast cell migration. *Molecular Medicine Reports*.

[15] Noguchi T., Wang L. L., Welsh D. K. (2013). Fibroblast PER2 circadian rhythmicity depends on cell density. *Journal of Biological Rhythms*.

[16] Yusko E. C., Asbury C. L. (2014). Force is a signal that cells cannot ignore. *Molecular Biology of the Cell*.

[17] Tong L., Yin L., Dan L., Fang P., Zhou L. (2009). Bioinformatics flow characteristics of acupuncture meridians and acupoints. *Chinese Medicine Modern Distance Education of China*.

[18] Zhang X., Cai D., Wang Y., Huang S. (2017). Effect of fibroblasts stimulated by acupuncture and mechanical stress. *Journal of Basic Chinese Medicine*.

[19] Chen B., Luo Y., Jin C., Feng X., Ling F. (2007). Comparative study on effects of static pressure stimulation on release of PGE_2_ and IL-6 in fibroblasts in the rat “Zusanli” and its adjacent areas. *Zhongguo Zhen Jiu*.

[20] Estes J. E., Selden L. A., Kinosian H. J., Gershman L. C. (1992). Tightly-bound divalent cation of actin. *Journal of Muscle Research and Cell Motility*.

[21] Chen M., Li A. (1997). Advances in research on actin, actin-binding protein and cell movement. *Life Sciences*.

[22] Yang W., Zhang X., Guo Y. (2009). The response of metabolism and tubulin of cardiac myocytes in vitro to mechanical stretch. *Chinese Journal of Biomedical Engineering*.

[23] Vogl T., Ludwig S., Goebeler M. (2004). MRP8 and MRP14 control microtubule reorganization during transendothelial migration of phagocytes. *Blood*.

[24] Yao Y., Liang W., Ye D. (2014). Cytoskeleton and mechanical signal transduction. *Chinese Journal of Tissue Engineering Research*.

[25] Nakamichi K., Saiki M., Kitani H. (2007). Roles of NF-*κ*B and MAPK signaling pathways in morphological and cytoskeletal responses of microglia to double-stranded RNA. *Neuroscience Letters*.

[26] Park E., Kang S., Lee Y. (2008). Integrity of the cortical actin ring is required for activation of the PI3K/Akt and p38 MAPK signaling pathways in redifferentiation of chondrocytes on chitosan. *Cell Biology International*.

[27] Qu F., Cui Y., Zeng J. (2019). Acupuncture induces adenosine in fibroblasts through energy metabolism and promotes proliferation by activating MAPK signaling pathway via adenosine 3 receptor. *Journal of Cellular Physiology*.

